# Prospective analysis of pain expectancy and experience during MR-fusion prostate biopsy: does reality match patients’ expectancy?

**DOI:** 10.1007/s00345-022-04083-3

**Published:** 2022-07-14

**Authors:** Philipp Krausewitz, Helene Schmeller, Julian Luetkens, Darius Dabir, Jörg Ellinger, Manuel Ritter, Rupert Conrad

**Affiliations:** 1 Department of Urology and Pediatric Urology, University Hospital Bonn, Bonn, Germany; 2 Department of Diagnostic and Interventional Radiology, University Hospital Bonn, Bonn, Germany; 3 Department of Psychosomatic Medicine and Psychotherapy, University Hospital Bonn, Bonn, Germany; 4 Department of Psychosomatic Medicine and Psychotherapy, University Hospital Muenster, Muenster, Germany; 5 Department of Urology and Pediatric Urology, University Hospital Bonn, Bonn, Germany

**Keywords:** Pain expectancy, Pain, Prostate biopsy, Anxiety, Perceived stress, MRI

## Abstract

**Purpose:**

Multiparametric magnetic resonance imaging fusion targeted prostate biopsy (MR-TB) has emerged to the biopsy technique of choice for evaluation of patients with suspected prostate cancer (PCA). The study aimed to determine expected and experienced pain during MR-TB depending on patients’ psychological state.

**Methods:**

We prospectively enrolled 108 men with suspicion of PCA who underwent MR-TB. All patients completed self-reported validated questionnaires assessing pain, stress, self-efficacy, anxiety and study-specific questionnaires on expected and experienced pain before, during and after MR-TB. Patient characteristics and survey scores were obtained.

**Results:**

Overall, pain levels during MR-TB were low (mean 2.8/10 ± 2.5 Numerical Rating Scale, NRS). 10/86 (11.6%) participants reported severe pain (≥ 7/10 NRS). Pain correlated significantly with anxiety (*r* = 0.42), stress (*r* = 0.22) and pain expectancy (*r* = 0.58). High self-efficacy did not show increased pain resilience. Participants anticipated more pain than experienced during each step of MR-TB with significant differences concerning local anesthesia and core sampling (both *p* < 0.001), among others. Expectancy and actual pain did not match regarding severity and impact of the total amount of cores taken (*p* < 0.05). Independent predictors of increased pain at biopsy were prostate volume > 50 ml (*p* = 0.0179) and expected pain during rectal manipulation (*p* < 0.001).

**Conclusion:**

Pain during MR-TB can be positively influenced by reducing men’s anxiety, stress and pain expectancy. To meet the needs of the audience, clinicians should address concrete pain levels of each procedural step and consider special treatment for patients with prostate volume > 50 ml and men reporting on increased rectal sensitivity.

**Supplementary Information:**

The online version contains supplementary material available at 10.1007/s00345-022-04083-3.

## Introduction

Despite major advances in imaging techniques, in particular multiparametric magnetic imaging (MRI), prostate biopsy is still essential in the diagnosis of prostate cancer (PCA). Guidelines recommend the combined approach of both, systematic biopsy (SB) and MRI-targeted biopsy (combined MRI-fusion biopsy, MR-TB) [1]. Yet, fear and distress of biopsy-related pain are frequent in men, thus delaying or omitting necessary medical examinations [2, 3]. MR-TB led to a higher total core number, which is mentioned as a reason for increased anxiety and discomfort [4, 5]. On the other side, a MRI-only pathway has the potential to reduce unnecessary biopsies and hence, reduce pain and anxiety in men [6]. Bearing in mind that interventional risks of prostate biopsy are low with serious adverse events occurring in approximately 1% of all cases, reported anxiety levels seem disproportionately high [7, 8]. This might be explained by the occurrence of multiple low-grade adverse events resulting in the dissemination of biased and inaccurate information to patients [2]. Expectation of pain and anxiety are interdependent and both predictive for pain-levels. Patients who reported more anxiety and higher pain expectancy experienced more pain during the procedure [5, 9, 10]. An adequate preoperative medical consultation including education and guidance of biopsy risks may reduce anxiety and pain levels, health care contacts and distress regardless of the occurrence of adverse events [11].

Preinterventional medical education mostly focusses on adverse events like infectious and bleeding complications. Men’s pain experience and psychological wellbeing during the procedure is rarely discussed. To better understand patients’ needs, we investigated expected and experienced pain during different steps of MR-TB and simultaneously determined self-efficacy, perceived stress and anxiety of men to develop new strategies to increase patients’ resilience.

## Design and methods

### Design/ethical approval

The prospective, interventional study was approved by the Ethics Committee at the Medical Faculty of the Rheinische Friedrich-Wilhelms University, Bonn (No. 307/20) and registered in the German Clinical Study Register (DRKS00022361;2020). Study conduct was in accordance with local regulations and laws, the ethical principles of the Declaration of Helsinki, and the principles of good clinical practice.

### Participants

Men with suspected PCA, aged 45–85 years, awaiting MR-TB were screened for eligibility from August 2020 to April 2021. Suspicion of localized PCA was based on elevated Prostate-specific Antigen (PSA), abnormal digital rectal examination (DRE) and/or abnormal findings on transrectal ultrasound (US). Both, biopsy naïve and previously biopsied men were included. Patients with known psychiatric disease, abuse of medication or drugs and contraindications for MRI were excluded. Out of 175 men attending, 146 participated after written informed consent was obtained (83% response rate). Reasons for not participating were lack of time and/or concentration, language barriers, or being overwhelmed by the number of questions (*n* = 29). 38 patients did not undergo MR-TB because of unsuspicious MRI results, defined as Prostate Imaging Reporting & Data System (PI-RADS) ≤ 2 [12] and/or patients decision to perform PSA follow-up despite MRI result. 108 men were included in analysis (Supplementary Fig. 1).

### Counselling process

During the first visit for MRI acquisition (T0) participants were provided with additional written instructions and information concerning MR-TB for self-study and preparation for the upcoming doctor’s appointment (Supplementary Appendix 1). In between T0 and T1 patients were additionally informed in writing and orally about risks, benefits, side effects and conduct of prostate biopsy in a 30-min doctor’s consultation as part of the clinical routine. Informed consent was obtained by or under supervision of two highly trained urologists who performed the biopsy procedure later.

### Prostate biopsy

Two experienced urologists (> 500 MR-TB) performed standardized 12-core SB and MRI-targeted biopsies of suspicious lesions (PI-RADS ≥ 3) in left lateral decubitus under local anesthesia consisting of both, intra-rectal lidocaine gel (11 ml) and nerve block (10 ml Mecain 1%, 10 mg/ml) injected at the dorsobasal prostatic capsule. All patients received intravenous antibiotic prophylaxis and povidone-iodine cleansing before the biopsy. MR-TB was performed using a software-assisted fusion technique (KOELIS Trinity^®^). No other analgesic or anxiolytic measures were used before the procedure and no pictorial or audiological distraction tactics were applied during the biopsy.

### Validated questionnaires

Participants completed validated questionnaires at the day of MRI acquisition (T0) directly before (T1) and after the procedure (T2). The Brief Pain Inventory (BPI) [13], the Perceived Stress Scale (PSS) [14], the General Self-Efficacy Scale (GSES) [15], and the State-Trait Anxiety Inventory (STAI) [15] were used to evaluate pain, distress, self-efficacy, and anxiety, respectively (Supplementary Table 1). Hereby, the BPI was adjusted to two hours in order to assess the pain during MR-TB accurately.

In addition, two questionnaires were created by modifying questions from other validated pain-related questionnaires based on the widely used and validated 11-point numerical rating scale (NRS; 0 = “no pain,” 10 = “most severe pain”) to assess pain at different steps of the biopsy procedure [4, 16–18]. Questionnaires were adjusted to enable a detailed investigation of pain experience (T2) and expectations (T0, T1) at each step of MRT-TB: DRE, rectal cleansing, probe insertion, application of the local anesthetic, placement and movement of US probe during fusion of US/MRI data, core sampling. Moreover, participants rated on a standardized, previously used 5-point scale whether the number of cores will have or had a strong influence on their pain during biopsy [9]. (Supplementary Appendix 2).

### Statistical analyses

Data were analyzed with “IBM SPSS Statistics,” version 26. Differences were detected using the *T*-test for independent samples (comparison of means, interval scaled) or chi-square tests (comparison of frequencies, nominal scaled). Cohen’s *d* as a measure of effect size was calculated when significant differences between means were present. Correlations between patient data were calculated according to Pearson. To describe the amount of explained variance in linear regression the corrected *R*^2^ was used. Effect size, correlations and regression analysis were interpreted according to Cohen [19]. *P* values of ≤ 0.05 were considered statistically significant for all models.

## Results

PCA was detected in 66.7% of men with on average 14.7 ± 2.6 biopsy cores taken. MRI target lesions were allocated in the anterior or transitional zone of the prostate in 44/108 (40.7%) patients. The detailed patients’ characteristics are provided in the Supplementary Table 2.

**Fig. 1:**
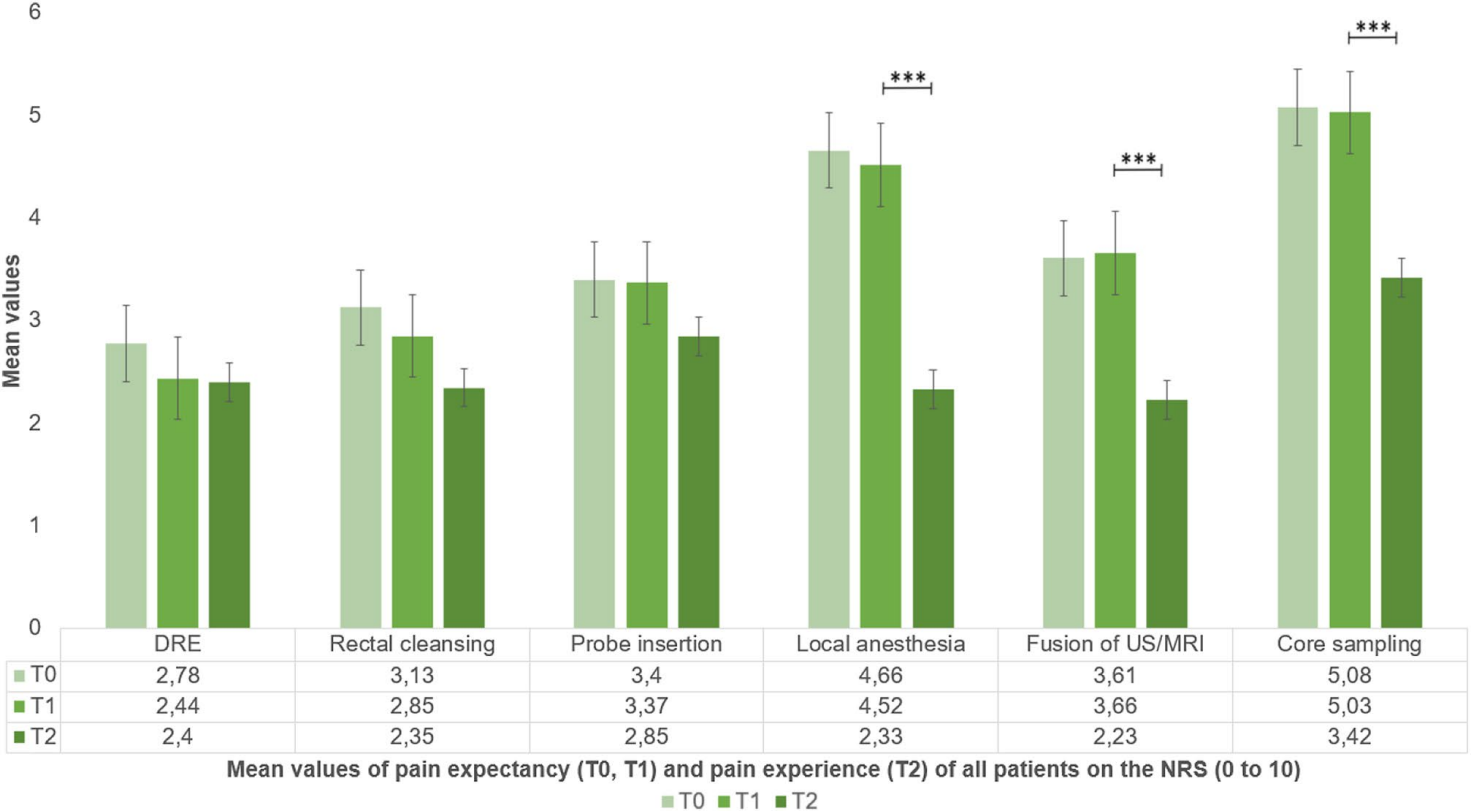
Expected and experienced pain during individual steps of MRI-fusion biopsy. T0: first study visit before medical consultation on prostate biopsy during MRI acquisition. T1: second study visit directly before prostate biopsy. T2: third study visit directly after prostate biopsy. *NRS* numerical Rating Scale for pain

Most participants scored in the lower range for pain at T2 (2.8/10 NRS), whereas scores on expected pain during biopsy (T0: 22.4/60; T1: 22.0/60) were in the middle range (Supplementary Table 3). Hence, expected pain scores (T0, T1) were higher than experienced pain scores (T2). Significant differences were found for the application of local anesthesia (Δ 2.19 NRS), the US/MRI fusion (Δ 1.36 NRS), and core sampling (Δ 1.61 NRS), all *p* < 0.001. Similar differences were shown for the period T0/T2 (all *p* < 0.001; Fig. 1, Supplementary Table 4). Overall pain expectancy before the biopsy at T0 and T1 were comparable (BPI 22.4/60 ± 12.7 (T0) and BPI 22.0/60 ± 9.9 (T1); Supplementary Table 3). Patients reported low pain on average at all visits (T0, T1 and T2), with a moderate increase of 13% from baseline to T2. Immediately after the biopsy, the proportion of men suffering from severe pain increased from 4.1 to 11.6%. However, regarding the current pain level after the biopsy, only 4.7% of patients experienced severe pain (Supplementary Fig. 2).

Most men expected severe pain (≥ 7/10 on NRS) during core sampling and application of local anesthesia (T0: 35.1%, T1: 25.0% and T0: 27.8%, T1: 23.0%, respectively). After the biopsy, core sampling was accordingly rated the most painful part of the biopsy (T2: 3.4/10 NRS), but other preparatory steps (DRE, rectal cleansing and probe insertion) were rated more painful than the application of local anesthesia (Fig. 1). The proportion of men who experienced severe pain during core sampling was only 12.1% with a similar proportion of 11% experiencing probe insertion as very painful. Doctor’s counselling before the biopsy did not significantly reduce overall expectations of severe pain and expectations of pain at individual steps of the biopsy (ΔT0/T1). However, the proportion of patients who expected the most severe pain during different steps of biopsy changed after medical consultation: We determined the largest differences during probe insertion (Δ − 11.7%), DRE (Δ − 11.2%), core sampling (Δ − 10.1%) and rectal cleansing (Δ − 9.0%), respectively (Supplementary Fig. 3).

Patients who expected more pain during rectal manipulation including DRE, rectal cleansing and probe insertion also experienced significantly more pain (all *p* < 0.001), showing a strong correlation between anticipated and experienced pain (for all *r* > 0.50). In addition, pain experience at DRE, rectal cleansing and probe insertion also correlated strongly (for all *r* > 0.70 (Supplement Table 5).

Moreover, we determined a strong correlation between pain perception and experience during MR-TB in general (T0: *r* = 0.50, T1: *r* = 0.58). In linear regression analysis, expected pain explained a medium to large proportion of the variance in experienced pain ranging from 23.8% (T0) to 32.8% (T1). Significant correlations were also shown for experienced pain and anxiety (T2: *r* = 0.42) as well as perceived stress (T2: *r* = 0.22). Perceived stress correlated with expected (T0: *p* < 0.05, T1: *p* < 0.01) and experienced (T2: *p* < 0.05) pain during biopsy and explained a small proportion of the pain experience. Self-efficacy did affect neither pain expectation nor experience, even if self-efficacy showed a strong correlation with trait anxiety (*r* = 0.35) and perceived stress (*r* = 0.32, Table 1).General anxiety explained a small proportion of additional variance in pain expectancy and sensation. (Supplementary Table 5).

**Table 1:** Intercorrelations (by Pearson) between pain and predictors

	1	2	3	4	5	6	7	8	9	10	11
1.BPI (T0)	–										
2.BPI (T1)	0.253*	–									
3.BPI (T2)	0.037	0.413***	–								
4.Expected pain (T0)	0.025	0.095	0.274*	–							
5.Expected pain (T1)	0.115	0.159	0.270*	0.605***	–						
6.Experienced pain (T2)	0.106	0.066	0.490***	0.498***	0.580***	–					
7.STAI-T (T0)	0.228*	0.054	0.207	0.077	0.278*	0.287**	–				
8.STAI-S (T0)	0.070	− 0.017	0.158	0.043	0.207	0.034	0.595**	–			
9.STAI-S (T1)	− 0.040	0.190	0.245*	0.135	0.355**	0.174	0.506***	0.526***	–		
10.STAI-S (T2)	0.073	0.053	0.288**	0.036	0.378***	0.416***	0.686***	0.562***	0.685***	–	
11.PSS (T0)	0.178	0.112	0.132	0.242*	0.315**	0.222*	0.696***	0.497***	0.289**	0.403***	–
12.GSES (T0)	− 0.173	0.022	− 0.104	− 0.087	− 0.195	− 0.119	− 0.351***	− 0.321**	− 0.180	− 0.262*	− 0.323**

T0: first study visit before medical consultation on prostate biopsy during MRI acquisition. T1: second study visit directly before prostate biopsy. T2: third study visit directly after prostate biopsy

*BPI* Brief Pain Inventory, no. 3: “Most severe pain in the last 2 h”, *STAI* State-Trait Anxiety Inventory (T Trait, S State), *PSS* Perceived Stress Scale, *GSES* General Self-Efficacy Scale

*p < 0.05, **p < 0.01, ***p < 0.001. *r* = 0.10 small, *r* = 0.30 medium, *r* = 0.50 large correlation

Number of cores, first versus repeated biopsy, operator, patient age, postinterventional complications, disease status and target location did not independently predict pain at biopsy. The only independent predictor of increased levels of pain during prostate biopsy was prostate size > 50 ml. A detailed description of the subgroup analysis is provided in Supplementary material.

## Discussion

MR-TB consists of several steps that are more or less painful for patients. A better understanding of the pain process during the biopsy could help to optimize patients’ experience. For the first time, we assessed pain perception and experience at each step of MR-TB both before (T0, T1) and after the procedure (T2) and simultaneously assessed self-efficacy, perceived stress and anxiety to develop new strategies to improve tolerance of MR-TB.

Contrary to their expectations, most men scored in the lower range for pain (2.8/10 NRS) with only 11.6% stating severe pain. These results are in line with previous series investigating transrectal and transperineal biopsy [4, 9, 18, 20]. As expected, application of local anesthesia in the transrectal approach causes less pain (2.3/10 vs. 3.9/10 NRS) [18]. Moreover, the detected proportion of men suffering from severe pain (11.6% during biopsy and 4.7% after biopsy) appears markedly smaller compared to previous series (16–29%) [4, 9, 17], which is best explained by the two-folded anesthesia and short procedure time [21].

The study also elaborated previously mentioned significant correlations between biopsy-related pain and predictors like anxiety and stress [4, 9, 20]. Hence, in addition to pain control, urologists’ obligation is to reduce anxiety and stress during preinterventional counselling and MR-TB itself.

Another key finding of our analysis is that men expect more pain than they do ultimately experience and both correlate significantly with each other (*p* < 0.001). Expected pain explained a medium to large proportion of the variance of experienced pain. It is therefore important to create realistic expectations of pain during informed consent.

Interestingly, medical consultation at T1 neither changed overall nor specific pain expectancy in our cohort. It only influenced the proportion of patients who expected severe pain at certain steps of the biopsy. Nevertheless, adjustment of expectations was insufficient regarding an appropriate clarification of pain for each stage. In addition, falsely favorable expectations were raised for rectal cleansing, local anesthesia and DRE. In keeping with this finding, Wade et al. showed that patients, who felt inadequately prepared for prostate biopsy, experienced side effects as more problematic afterwards, felt more anxious and contacted medical professionals more often [11]. Hence, patients should be informed evidence-based about pain levels of previous patient cohorts to form realistic expectations and consecutively improve their well-being during MR-TB.

We identified target groups who demand a special counselling process. Pain experience and expectations during different steps of rectal manipulation (DRE, rectal cleansing and probe insertion) correlated strongly, indicating a subgroup of men with increased rectal sensitivity, which needs to be considered during preparation and conduct of MR-TB, for example placement of additional lubrication or anesthetic. Furthermore, patients with large prostates (≥ 50 ml) need to be informed to expect more pain to be able to optimize pain treatment.

Our results corroborate prior prospective series showing that in patients undergoing repeated biopsies the discrepancy between pain expectations and experience decreases, whereas the degree of actual pain or anxiety does not [4, 9]. Nevertheless, their pain expectancy still exceeded their experience calling for a comprehensive preinterventional education.

Although we confirmed previous findings showing no correlation between the number of cores and pain severity, about 40% of men feared a negative impact of an increased amount of samples on pain [22, 23]. Hence, the number of cores is another relevant topic to discuss during informed consent, but is possibly not decisive for the pain experience. This finding significantly undermines the argument of increased wellbeing by choosing the MRI-only pathway.

Our study is not without limitations. First, beside the BPI, we used institutional modified questionnaires based on the validated 11-point numerical rating scale for interpersonal pain assessment [4, 16–18] during each step of the biopsy, as no suitable validated questionnaires were available. Second, we did not assess the willingness to undergo prostate biopsy. Therefore, we cannot exclude a selection bias of men particularly willing to undergo repeated biopsy due to rather positive prior expectations or anxious men refusing medical intervention despite prostate cancer. However, such bias seems to have a rather small impact, as 95% of men reported independently after their experience that they would undergo another biopsy, if their doctor would recommend it [17].

Despite this, we believe that our study provided clinically relevant insights into patient psychological wellbeing during MR-TB including pain, anxiety and stress. Especially, to date no other study showing absolute and relative discrepancies between patients’ pain perception and experience during individual steps of MR-TB is available.

## Conclusion

Pain during MR-TB can be positively influenced by reducing men’s anxiety, stress and pain expectancy. To meet the needs of the audience, clinicians should address concrete pain levels of each procedural step and consider special treatment for patients with prostate volume > 50 ml and men reporting on increased rectal sensitivity.

## Supplementary Information

Below is the link to the electronic supplementary material.

Supplementary file1 (DOCX 957 KB)
