# Supplementary material for: Prospective analysis of pain expectancy and experience during MR-fusion prostate biopsy: does reality match patients’ expectancy?
Source: World J Urol. 2022 Jul 14;40(9):2239–44. doi: 10.1007/s00345-022-04083-3 (PMC9427866; doi:10.1007/s00345-022-04083-3)
Supplement: Supplementary file 1 — Supplementary file1 (DOCX 957 KB) [file 345_2022_4083_MOESM1_ESM.docx]

**Prospective analysis of pain expectancy and experience during MR-fusion prostate biopsy**

**Does reality match patients’ expectancy?**

P. Krausewitz^1^, H. Schmeller^1^, J. Luetkens^2^, D. Dabir^2^, J. Ellinger^1^, M. Ritter^1^, R. Conrad^3^

^1^University Hospital Bonn, Department of Urology, Bonn, Germany

^2^University Hospital Bonn, Department of Diagnostic and Interventional Radiology, Bonn, Germany

^3^University Hospital Bonn, Department of Psychosomatic Medicine and Psychotherapy, Bonn, Germany

Address for correspondence: Philipp Krausewitz, M.D.

Department of Urology and Pediatric Urology,

University Hospital Bonn

Email: [Philipp.krausewitz@ukbonn.de](mailto:Philipp.krausewitz@ukbonn.de)

Tel.: +4915118853551

Orcid-ID 0000-0002-8213-9975

**Supplementary Material**

**Supplementary Fig. 1 Consort Trial Flow Chart**

Follow-Up: T1

Follow-Up: T2

Analysis

Data were collected (n=45)

Lost to follow-up (n=9): General anesthesia n=3, study rejected: n=6

Analysed (n=54)

Data were collected (n=46)

Lost to follow-up (n=8): General anesthesia n=3, study rejected: n=5

)

Data were collected (n=48)

Lost to follow-up (n=6): General anesthesia n=3, study rejected: n=3

Data were collected (n=45)

Lost to follow-up (n=9): General anesthesia n=3, study rejected: n=6

**Emotional-based information**

Received allocated intervention (n=54)

Data were collected (n=54)

Analysed (n=54)

**Facts-based information**

Received allocated intervention (n=54)

Data were collected (n=54)

Assessed for eligibility (n=175)

Excluded (n=67)

♦  Not meeting inclusion criteria (n=38)

♦  Declined to participate (n=18)

♦  Other reasons (n=11)

Randomized (n=108)

Allocation: T0

Enrollment

The consort trial chart shows the allocation of the study participants through the trial according to the criteria recommended in the CONSORT extension guideline. The influence of content and style of written medical information will be analysed in another analysis.

**Supplementary Fig. 2 Pain expectancy and experience before and after MRI-fusion biopsy measured by BPI**

T0: first study visit before medical consultation on prostate biopsy during MRI acquisition. T1: second study visit directly before prostate biopsy. T2: third study visit directly after prostate biopsy. NRS, Numerical Rating Scale for pain is a is unidimensional measure of pain intensity in adults in which a respondent selects a whole number (0 to 10 integers) that best reflects the intensity of their pain.

**Supplementary Fig. 3** **Expected and experienced severe pain during each step of MRI-fusion biopsy and reported impact on pain by total number of cores taken**

T0: first study visit before medical consultation on prostate biopsy during MRI acquisition. T1: second study visit directly before prostate biopsy. T2: third study visit directly after prostate biopsy. NRS, Numerical Rating Scale for pain.

**Supplementary Table 1 Intervention scheme**

|  | **Visit 1 (T0) Day -50** | **Visit 2 (T1) Day 0**  **Before biopsy** | **Visit 2 Day 0 MRI fusion biopsy** | **Visit 3 (T2) Day 0 After biopsy** |
| --- | --- | --- | --- | --- |
| **Acquisition of MRI of the prostate** | **√** |  |  |  |
| **In-/Exclusion Criteria** | **√** |  |  |  |
| **Informed Consent** | **√** |  |  |  |
| **General Self Efficacy Scale** | **√** |  |  |  |
| **Perceived Stress Scale** | **√** |  |  |  |
| **State Trait Anxiety Inventory (State)** | **√** | **√** |  | **√** |
| **State Trait Anxiety Inventory (Trait)** | **√** |  |  |  |
| **T0-Questionnaire regarding additional consultation** | **√** |  |  |  |
| **T1-Questionnaire regarding additional consultation** |  | **√** |  |  |
| **Experienced Pain Questionnaire** |  | **√** |  |  |
| **Combined MRI-fusion biopsy** |  |  | **√** |  |
| **T2-Questionnaire regarding additional consultation** |  |  |  | **√** |
| **Experienced Pain Questionnaire** |  |  |  | **√** |

T0: first study visit before medical consultation on prostate biopsy during MRI acquisition; T1: second study visit directly before prostate biopsy; T2: third study visit directly after prostate biopsy. MRI, multiparametric magnetic imaging of the prostate.

**Supplementary Table 2** **Descriptive statistics of clinical measures**

|  | All men (n=108) |
| --- | --- |
| Age (years) | 66.2 ± 8.53 |
| PSA (ng/ml) | 8.2 ± 5.9 |
| Prostate volume (ml) | 57.6 ± 32.2 |
| Abnormal DRE (%) | 37.4 |
| Abnormal US (%) | 31.1 |
| PI-RADS ≥ 3 (%) | 77.8 |
| Anterior MRI target lesion (%) | 40.7 |
| Re-Biopsy (%) | 28.7 |
| Active Surveillance (%) | 2.8 |
| Waiting period (days) | 18 ± 15.1 |
| Total number of cores | 14.7 ± 2.6 |
| Time of biopsy procedure (min) | 8.9 ± 3.9 |
| PCA detection (%) | 66.7 |
| csPCA detection (%) | 56.4 |

Re-Biopsy, Repeated biopsy; PCA, Prostate cancer; csPCA, clinical significant prostate cancer defined as Gleason ≥ 3+4; PSA, prostate specific antigen; DRE, digital rectal examination; US, transrectal ultrasound; PI-RADS, The Prostate Imaging - Reporting and Data System Version 2 (PI-RADS™ v2.1); anterior MRI target lesion, PCA suspicious lesions (PI-RADS ≥ 3) in the anterior or transitional zones defined by the PI-RADS™ v2.1 prostate sector map

**Supplementary Table 3** **Descriptive statistics for predictor and outcome variables**

|  | n | Test Range | Sample Range | M | SD |
| --- | --- | --- | --- | --- | --- |
| BPI (T0) | 98 | 0-10 | 0-8 | 1.5 | 2.2 |
| BPI (T1) | 89 | 0-10 | 0-9 | 0.8 | 1.5 |
| BPI (T2) | 86 | 0-10 | 0-10 | 2.8 | 2.5 |
| Expected pain (T0) | 91 | 0-60 | 0-51 | 22.4 | 12.7 |
| Expected pain (T1) | 86 | 0-60 | 4-45 | 22.0 | 9.9 |
| Experienced pain (T2) | 88 | 0-60 | 0-54 | 15.7 | 12.1 |
| STAI-T (T0) | 96 | 20-80 | 21-60 | 34.7 | 8.2 |
| STAI-S (T0) | 101 | 20-80 | 20-72 | 38.9 | 9.9 |
| STAI-S (T1) | 90 | 20-80 | 20-70 | 43.7 | 9.9 |
| STAI-S (T2) | 88 | 20-80 | 20-70 | 37.4 | 10.3 |
| PSS (T0) | 104 | 0-40 | 4-30 | 14.3 | 5.1 |
| GSES (T0) | 101 | 10-40 | 22-40 | 31.4 | 3.7 |

M: Mean values. SD: Standard deviation. T0: first study visit before medical consultation on prostate biopsy during MRI acquisition. T1: second study visit directly before prostate biopsy. T2: third study visit directly after prostate biopsy. BPI, Brief Pain Inventory, no. 3: "Most severe pain in the last 2 hours"; STAI, State-Trait Anxiety Inventory (T Trait, S State); PSS, Perceived Stress Scale; GSES, General Self-Efficacy Scale.

**Supplementary Table 4** **Descriptive statistics of** **pain expectancy and experience during individual steps of MRI-fusion biopsy**

|  | N | Test Range | Sample Range | M | SD |
| --- | --- | --- | --- | --- | --- |
| **Expected pain (T0)** | |  |  |  |  |
| DRE | 97 | 0-10 | 0-10 | 2.8 | 2.6 |
| Rectal cleansing | 97 | 0-10 | 0-9 | 3.1 | 2.4 |
| Probe insertion | 98 | 0-10 | 0-10 | 3.4 | 2.7 |
| Local anesthesia | 97 | 0-10 | 0-10 | 3.7 | 2.6 |
| Fusion of US/MRI | 96 | 0-10 | 0-10 | 3.6 | 2.4 |
| Core sampling | 97 | 0-10 | 0-10 | 5.1 | 2.6 |
| **Expected pain (T1)** | |  |  |  |  |
| DRE | 89 | 0-10 | 0-8 | 2.4 | 1.9 |
| Rectal cleansing | 89 | 0-10 | 0-8 | 2.9 | 1.9 |
| Probe insertion | 89 | 0-10 | 0-8 | 3.4 | 1.9 |
| Local anesthesia | 87 | 0-10 | 0-9 | 4.5 | 2.3 |
| Fusion of US/MRI | 87 | 0-10 | 0-8 | 3.7 | 2.0 |
| Core sampling | 88 | 0-10 | 1-10 | 5.0 | 2.2 |
| **Experienced pain (T2)** | |  |  |  |  |
| DRE | 90 | 0-10 | 0-9 | 2.4 | 2.2 |
| Rectal cleansing | 89 | 0-10 | 0-10 | 2.5 | 2.4 |
| Probe insertion | 91 | 0-10 | 0-10 | 2.9 | 2.6 |
| Local anesthesia | 91 | 0-10 | 0-10 | 2.3 | 2.4 |
| Fusion of US/MRI | 91 | 0-10 | 0-9 | 2.2 | 2.1 |
| Core sampling | 91 | 0-10 | 0-10 | 3.4 | 2.4 |

M: Mean values. SD: Standard deviation. T0: first study visit before medical consultation on prostate biopsy during MRI acquisition. T1: second study visit directly before prostate biopsy. T2: third study visit directly after prostate biopsy. DRE, digital rectal examination; US, transrectal Ultrasound; MRI, multiparametric magnetic resonance imaging of the prostate

**Supplementary Table 5 Intercorrelations (by Pearson) between pain expectancy and experience of rectal manipulation**

|  | 1 | 2 | 3 | 4 | 5 | 6 | 7 | 8 |
| --- | --- | --- | --- | --- | --- | --- | --- | --- |
| 1. Expected pain T0 DRE | - |  |  |  |  |  |  |  |
| 1. Expected pain T1 DRE | **,421***** | **-** |  |  |  |  |  |  |
| 1. Experienced pain T2 DRE | **,392***** | **,508***** | **-** |  |  |  |  |  |
| 1. Expected pain T0 Rectal cleansing | **,761***** | **,411***** | **,344**** | **-** |  |  |  |  |
| 1. Expected pain T1 Rectal cleansing | **,320**** | **,784***** | **,432***** | **,415***** | **-** |  |  |  |
| 1. Experienced pain T2 Rectal cleansing | **,291**** | **,482***** | **,712***** | **,398***** | **,591***** | **-** |  |  |
| 1. Expected pain T0 Probe insertion | **,776***** | **,390***** | **,329**** | **,848***** | **,362**** | **,347**** | **-** |  |
| 1. Expected pain T1 Probe insertion | **,410***** | **,733***** | **,562***** | **,445***** | **,701***** | **,512***** | **,459***** | **-** |
| 1. Experienced pain T2 Probe insertion | **,328**** | **,388***** | **,707***** | **,361**** | **,421***** | **,765***** | **,391***** | **,520***** |

*p < 0.05, ** p < 0.01, *** p < 0.001. |R| = .10 small, |R| = .30 medium, |R| = .50 large correlation.

T0: first study visit before medical consultation on prostate biopsy during MRI acquisition. T1: second study visit directly before prostate biopsy. T2: third study visit directly after prostate biopsy. DRE, Digital rectal examination.

**Supplementary Table 6** **Regression analysis of trait anxiety, stress, and self-efficacy on expected and experienced pain during MRI-fusion biopsy, and the role of expected pain on experienced pain during biopsy.**

|  | **Expected pain (T0)** | | | **Expected pain (T1)** | | | **Experienced pain (T2)** | | |
| --- | --- | --- | --- | --- | --- | --- | --- | --- | --- |
|  | β | F | Korr. R^2^ | β | F | Korr. R^2^ | β | F | Korr. R^2^ |
| **STAI-T** | .077 | .490 | -.006 | .278* | .597* | **.065** | .287** | 7.248** | **.071** |
| **PSS** | .242* | 5.342* | **.048** | .315** | 8.822** | **.0822** | .222* | 4.234* | **.037** |
| **GSES** | -.087 | .661 | -.004 | -.195 | 3.272 | .026 | -.119 | 1.200 | .014 |
| **Expected pain (T0)** | - | - | - | .605*** | 42.04*** | **.357** | .498*** | 24.710*** | **.238** |
| **Expected pain (T1)** | - | - | - | - | - | - | .580*** | 41.103*** | **.328** |

*p < 0.05, ** p < 0.01, *** p < 0.001. β Standardized beta values. F F-change. Corr R2 Corrected R-squared. R² = .02 low, R² = .13 medium, R² = .26 high variance resolution. T0: first study visit before medical consultation on prostate biopsy during MRI acquisition. T1: second study visit directly before prostate biopsy. T2: third study visit directly after prostate biopsy. Expected or experienced pain during biopsy (sum score). STAI State-Trait Anxiety Inventory, T Trait. PSS Perceived Stress Scale. GSES General Self-Efficacy Scale.

**Subgroup analysis**

**Impact of the operator:** Two highly experienced urologists performed standardized 12-core SB and MR-targeted biopsy in all patients (Operator 1: 61 cases; Operator 2: 47 cases). We found no significant differences between the two operators regarding overall pain experience and the most severe pain during the last 2h at time T2, i.e. the total biopsy process (BPI). However, two significant differences were shown in analysis looking at the individual biopsy steps. Pain experience during DRE and rectal cleansing with iodine solution were significantly higher in one group (p=0.029 and p=0.034, respectively). However, patients biopsied in this group expected significantly more pain (p<0.05) at each step (except image fusion) immediately before the biopsy (T1) and reported more pain in the last 2h before the biopsy (BPI T1). At T0, no differences concerning the patients' pain expectations or pain (BPI) could be stated.

**Impact of prostate volume:** No significant differences in pain expectancy (T0, T1 BPI) between patients with prostate volume ≥50ml and <50ml were determined. However, patients with a prostate volume ≥50ml experienced significantly more overall pain during the biopsy (T2 BPI; p = 0.017) and during the step of rectal cleansing with iodine solution (p = 0.021). No differences were found concerning pain levels at the other stages of the prostate biopsy process. Choosing the cut off 30ml prostate volume, no differences concerning pain expectancy and experience were determined at any time.

**Impact of complications:** Overall 12/108 men (11.1%) experienced complications within the time window of investigation (T0-T2). Postinterventional complications included: 9/108 (8.3%) patients with hematuria without the need for intervention (Clavien Dindo I); 3/108 patients (2.8%) feeling fainted without suffering from an actual syncope and without further need for intervention (Clavien Dindo I); and 1/108 patients (0.9%) suffering from acute urinary retention with the need of a short-term transurethral disposable catheter placement (Clavien Dindo IIIa).

No significant differences between patients with and without side effects concerning pain expectancy or experience were determined. Neither before the biopsy T0/T1 nor after the biopsy (BPI and experienced pain questionnaire T2). Patients suffering from more severe pain (≥7/10 NRS) during (11.7%) and after the biopsy (4.7%) experienced no side effects.

**Impact of MRI target lesion location:** MRI target lesions were allocated in the anterior or transitional zone in 44/108 (40.7%) patients. Patients with tumor suspicious anterior or transitional zone lesions of the prostate expected significantly more pain during probe insertion immediately before the biopsy (T1; p= 0.02) and confirmed these afterwards with reporting significantly more pain during probe insertion at T2; p= 0.02). However, for all other individual biopsy steps and overall pain during the procedure, no significant differences concerning pain expectations and experience were found in comparison with men who harbor posterior lesions. In addition, reported levels of severe pain (BPI) did not differ between the groups at any time.

**Impact of total core number:** Our study did not show a correlation between experienced pain during the biopsy overall and the number of cores taken. However, pain expectations were significantly higher (at time T0 with 39.4% of men and at time T1 with 40.2% of men expecting the total core number to have a strong effect on their pain during MR-TB) than experienced pain (after the biopsy (T2), the proportion shrunk to 25% of men); p<0.001 (**Supplementary** **Fig. 3**).

**Impact of biopsy history:**

Trait anxiety and state anxiety at different time points in the diagnostic process did not differ significantly between patients with first and repeat biopsy. However, patients who had a repeat biopsy tended to report more anxiety immediately before the biopsy (T1) (p = 0.061). Furthermore, there were no differences in overall pain experience measured by BPI between the groups. Comparing biopsy naïve men and patients facing re-biopsy we solely found significant differences concerning the frequency of pain expectancy (T1) and experience (T2) concerning the number of cores taken (p = 0.019 and p = 0.011, respectively). 32.8% of the men who underwent first time biopsy stated in advance that the number of cores will have a strong influence on their pain and 18.2% of these men confirmed it later. Whereas, among patients with repeated biopsies, as much as 60.9% expected a strong impact and 45.5% of those verified their assumption. The effect size was small (Phi -0.252 (T1) and -0.273 (T2).

**Supplementary Appendix 1**

**Supplementary Appendix 2**
